# Spermidine Attenuates High Glucose-Induced Oxidative Damage in Retinal Pigment Epithelial Cells by Inhibiting Production of ROS and NF-κB/NLRP3 Inflammasome Pathway

**DOI:** 10.3390/ijms241310550

**Published:** 2023-06-23

**Authors:** EunJin Bang, Cheol Park, Hyun Hwangbo, Jung-Hyun Shim, Sun-Hee Leem, Jin Won Hyun, Gi-Young Kim, Yung Hyun Choi

**Affiliations:** 1 Anti-Aging Research Center and Core-Facility Center for Tissue Regeneration, Dong-Eui University, Busan 47340, Republic of Korea; ejbang@deu.ac.kr (E.B.); hhyun@deu.ac.kr (H.H.); 2 Department Division of Basic Sciences, College of Liberal Studies, Dong-Eui University, Busan 47340, Republic of Korea; parkch@deu.ac.kr; 3 Department of Pharmacy, College of Pharmacy, Mokpo National University, Jeonnam 58554, Republic of Korea; s1004jh@gmail.com; 4 Department Biomedicine, Health & Life Convergence Sciences, BK21 Four, College of Pharmacy, Mokpo National University, Muan 58554, Republic of Korea; 5 Department of Biomedical Sciences, Dong-A University, Busan 49315, Republic of Korea; shleem@dau.ac.kr; 6 Department of Health Sciences, The Graduated of Dong-A University, Busan 49315, Republic of Korea; 7 Department of Biochemistry, College of Medicine, Jeju National University, Jeju 63243, Republic of Korea; jinwonh@jejunu.ac.kr; 8 Jeju Research Center for Natural Medicine, Jeju National University, Jeju 63243, Republic of Korea; 9 Department of Marine Life Sciences, Jeju National University, Jeju 63243, Republic of Korea; immunkim@jejunu.ac.kr; 10 Department of Biochemistry, College of Korean Medicine, Dong-Eui University, Busan 47227, Republic of Korea

**Keywords:** apoptosis, NF-κB, NLRP3 inflammasome, ROS, spermidine

## Abstract

Diabetic retinopathy (DR) is the leading cause of vision loss and a critical complication of diabetes with a very complex etiology. The build-up of reactive oxygen species (ROS) due to hyperglycemia is recognized as a primary risk factor for DR. Although spermidine, a naturally occurring polyamine, has been reported to have antioxidant effects, its effectiveness in DR has not yet been examined. Therefore, in this study, we investigated whether spermidine could inhibit high glucose (HG)-promoted oxidative stress in human retinal pigment epithelial (RPE) cells. The results demonstrated that spermidine notably attenuated cytotoxicity and apoptosis in HG-treated RPE ARPE-19 cells, which was related to the inhibition of mitochondrial ROS production. Under HG conditions, interleukin (IL)-1β and IL-18’s release levels were markedly increased, coupled with nuclear factor kappa B (NF-κB) signaling activation. However, spermidine counteracted the HG-induced effects. Moreover, the expression of nucleotide-binding oligomerization domain-like receptor (NLR) protein 3 (NLRP3) inflammasome multiprotein complex molecules, including TXNIP, NLRP3, ASC, and caspase-1, increased in hyperglycemic ARPE-19 cells, but spermidine reversed these molecular changes. Collectively, our findings demonstrate that spermidine can protect RPE cells from HG-caused injury by reducing ROS and NF-κB/NLRP3 inflammasome pathway activation, indicating that spermidine could be a potential therapeutic compound for DR treatment.

## 1. Introduction

As the prevalence of diabetes has escalated continuously in recent decades, the incidence of diabetes-related complications has also increased [1,2]. Diabetic retinopathy (DR), the most persistent and severe diabetes complication, is a critical cause of visual impairment and vision loss, and hyperglycemia is a pivotal cause of DR pathogenesis [3,4]. Chronic hyperglycemia causes damage to the retinal blood vessels, and the retina, particularly the retinal pigment epithelial (RPE) cells, is very vulnerable to a hyperglycemic environment [5,6]. RPE cells, which reside at the interface between the neural retina and choroidal capillaries, play an important role in visual function, and their dysfunction due to excessive circulating glucose levels is closely related to the pathology of DR [7,8].

Recent studies have revealed that oxidative and inflammatory stress caused by high glucose (HG) conditions critically contribute to the initiation and progression of DR. According to previous studies, oxidative stress, which causes reactive oxygen species (ROS) accumulation due to hyperglycemia, is a key factor in DR-associated RPE cell damage [9,10,11]. A large number of intracellular ROS triggers inflammation and leads to the induction and progression of chronic diseases, including DR. Although there are multiple sources of ROS within cells, the mitochondria are the central site of ROS generation, which directly stimulates pro-inflammatory cytokine secretion and promotes pathological conditions [12]. Under HG conditions, mitochondrial ROS levels also increase, resulting in the activation of a chronic pro-inflammatory state [13,14].

Previous studies have reported that the nucleotide-binding oligomerization domain-like receptor (NLR) protein 3 (NLRP3) inflammasome signaling pathway is involved in DR [15]. This pathway is a critical component of innate non-specific immunity that promotes inflammatory responses to remove pathogens from physiological systems. Abnormal activation of inflammasomes has been related to age-related diseases, for example, diabetes, in which the gene and protein levels of NLRP3 inflammasome multiprotein complex molecules, apoptosis-associated speck-like proteins containing a caspase recruitment domain (ASC), and caspase-1 are notably upregulated in DR patients [16,17]. The NLRP3 inflammasome pathway is initiated when pathogenic molecules, such as damage-associated or pathogen-associated molecular patterns, are perceived by host cells. In canonical signaling, NLRP3 inflammasome priming leads to the nuclear translocation of pro-inflammatory nuclear factor kappa B (NF-κB), increasing the expression of target proteins, including NLRP3, ASC, pro-caspase-1, pro-interleukin (IL)-1β, and pro-IL-18, resulting in inflammatory responses [18].

Spermidine is a natural polyamine compound present in many edible plants and all living organisms, and it is critically involved in the maintenance of cellular homeostasis. Spermidine is involved in various cellular functions, including DNA synthesis, differentiation, proliferation, and migration [19,20]. In addition to these cellular functions, spermidine plays a biological function in the alleviation of ROS. For example, spermidine directly scavenges hydrogen peroxide (H_2_O_2_) and hydroxyl radicals and exerts protective effects against DNA oxidation [21]. In addition, spermidine suppresses pro-inflammatory cytokine secretion by blocking NF-κB signaling pathway activation, which could potentially exert therapeutic effects on development of age-related diseases, such as osteoarthritis [22]. However, the beneficial effects of spermidine for oxidative stress and inflammation in hyperglycemic RPE cells and its mechanism of action remain unclear. The present study examines the potential beneficial restorative effect of spermidine on ROS-mediated NF-κB/NLRP3 inflammasome activation in HG-induced hyperglycemic RPE cells.

## 2. Results

### 2.1. Spermidine Reduced HG-Induced Decrease in Cell Viability and Apoptosis

**Figure 1:**
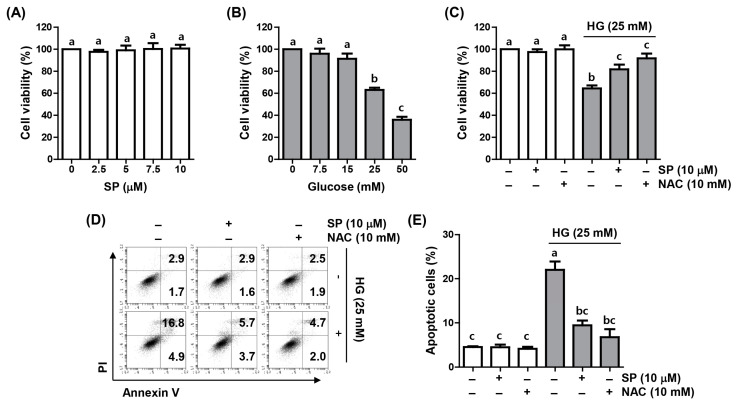
Inhibition of high glucose (HG)-induced cytotoxicity and apoptosis by spermidine in ARPE-19 cells. Cells were grown for 48 h in a medium with various concentrations of spermidine (**A**) and glucose (**B**) or pre-treated with spermidine (10 μM) or *N*-acetyl cysteine (NAC, 10 mM) for 1 h and cultured for an additional 48 h under HG (25 mM glucose) conditions (**C**–**E**). (**A**–**C**) Viable cells were measured using a 3-(4,5-dimethyl-2-thiazolyl)-2,5-diphenyltetrazolium bromide (MTT) assay. (**D**,**E**) Apoptotic cells were assessed using flow cytometry with Annexin V-fluorescein isothiocyanate (FITC)/propidium iodide (PI) staining. Representative images of flow cytometry (**D**) and quantification results (**E**) are shown. Numerical data are shown as the mean ± SD of three independent experiments. ^a–c^ Bars with different letters are significantly different at *p* < 0.05.

A 3-(4,5-dimethyl-2-thiazolyl)-2,5-diphenyltetrazolium bromide (MTT) assay was conducted to examine the effect of spermidine on the proliferation of ARPE-19 cells cultured under HG conditions. Compared to the control group, spermidine did not show a notable difference in cell viability at the range of concentrations used in the current study, but the HG group demonstrated decreased cell proliferation in a concentration-dependent manner (Figure 1A,B). Based on this result, the 25 mM glucose-treated group was set as the HG conditions, and the pre-treatment concentration of the spermidine was set as 10 μM. As demonstrated in Figure 1C, the 10 μM spermidine pre-treatment notably suppressed the cytotoxic effect of HG on ARPE-19 cell viability and reversed the decrease in cell proliferation induced by the HG treatment, similar to *N*-acetyl cysteine (NAC) pretreatment group, in which NAC is a potent antioxidant Next, we evaluated whether the inhibition of ARPE-19 cell viability by HG was related to the induction of apoptosis and whether spermidine could suppress it. As indicated in Figure 1D,E, flow cytometry results revealed a marked increase in apoptosis in cells cultured under HG conditions, but the upward trend in the HG-mediated apoptosis rate was notably attenuated in the presence of spermidine and NAC.

### 2.2. Spermidine Ameliorated HG-Induced Intracellular ROS Generation

**Figure 2:**
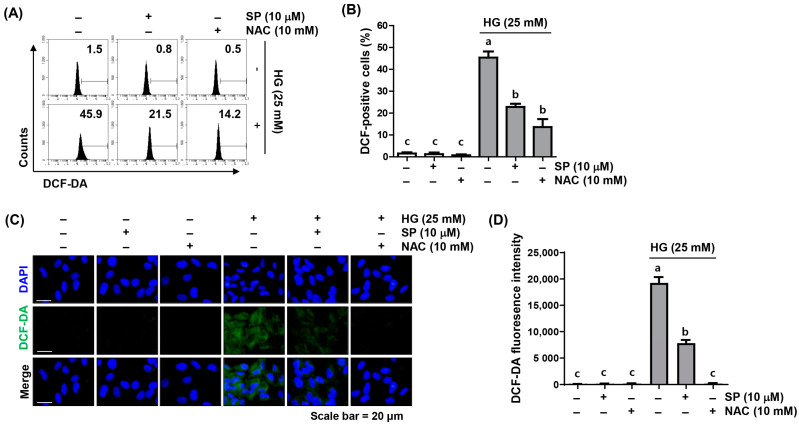
Suppression of HG-induced intracellular reactive oxygen species (ROS) accumulation by spermidine in ARPE-19 cells. Cells pre-treated with spermidine or NAC for 1 h were cultured in a growth culture medium containing HG for 1 h and then stained with 5,6-carboxy-2′,7′-dichlorodihydrofluorescein diacetate (DCF-DA). (**A**,**B**) Representative images of flow cytometry (**A**) and quantification results (**B**) are shown. (**C**,**D**) Representative images (green) of ROS within cells are demonstrated by fluorescence microscopy (200×). The nuclei (blue) were stained with 4′,6-diamidino-2-phenylindole DAPI, (**C**). The quantification results are shown (**D**). Numerical data are shown as the mean ± SD of three independent experiments. ^a–c^ Bars with different letters are significantly different at *p* < 0.05.

As decreased cell viability and the induction of apoptosis by HG stimulation were blocked, not only by spermidine but also by the ROS scavenger, NAC, we examined whether the beneficial effect of spermidine against HG-induced cytotoxicity was related to the mitigation of oxidative stress in ARPE-19 cells. In both the flow cytometry and fluorescence microscopy results using 5,6-carboxy-2′,7′-dichlorodihydrofluorescein diacetate (DCF-DA) staining, which reflects the amount of intracellular ROS generation, high ROS levels were detected in cells cultured under HG conditions; however, pre-treatment with spermidine or NAC prior to HG treatment reduced the ROS content by >50% compared to HG conditions (Figure 2), indicating that spermidine has potent ROS scavenging activity.

### 2.3. Spermidine Attenuated HG-Induced Mitochondrial ROS Generation

**Figure 3:**
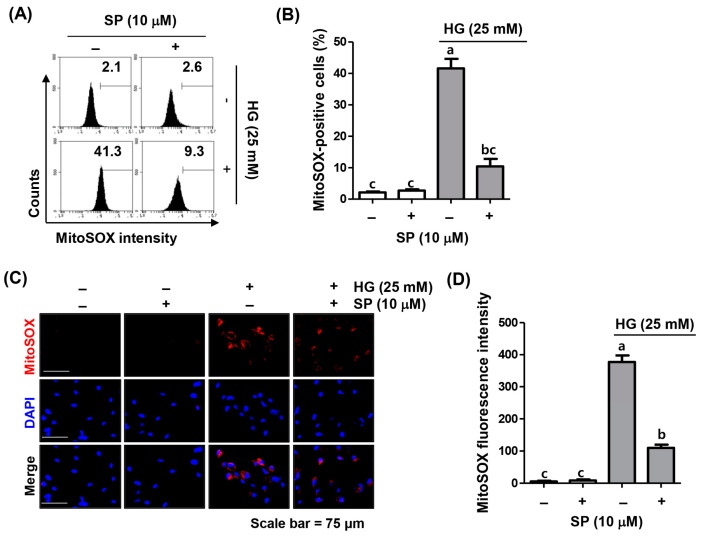
Suppression of HG-induced mitochondrial ROS accumulation by spermidine in ARPE-19 cells. Cells pre-treated with spermidine for 1 h were cultured in a growth culture medium including HG for 1 h and then stained with a MitoSOX red mitochondrial superoxide indicator. (**A**,**B**) Representative images of flow cytometry (**A**) and quantification results (**B**) are shown. (**C**,**D**) Representative images (red) of mitochondrial ROS visualized by fluorescence microscopy (400×). The nuclei (blue) were stained with DAPI (**C**). The quantification results (**D**) are shown. Numerical data are shown as the mean ± SD of three independent experiments. ^a–c^ Bars with different letters are significantly different at *p* < 0.05.

To determine whether mitochondria were the central source of the ROS under HG conditions, superoxide levels generated from the mitochondria were quantified by flow cytometry using MitoSOX staining. Similar to the DCF-DA results, the levels of the mitochondrial superoxide dramatically increased in cells cultured under HG conditions, which was further confirmed by the increased MitoSOX fluorescence intensity observed using fluorescence microscopy (Figure 3). However, spermidine dramatically abrogated HG-induced superoxide production, suggesting that spermidine relieved oxidative stress by blocking the production of the superoxide, a major ROS produced in the mitochondria under HG conditions.

### 2.4. Spermidine Alleviated HG-Induced Inflammatory Response

**Figure 4:**
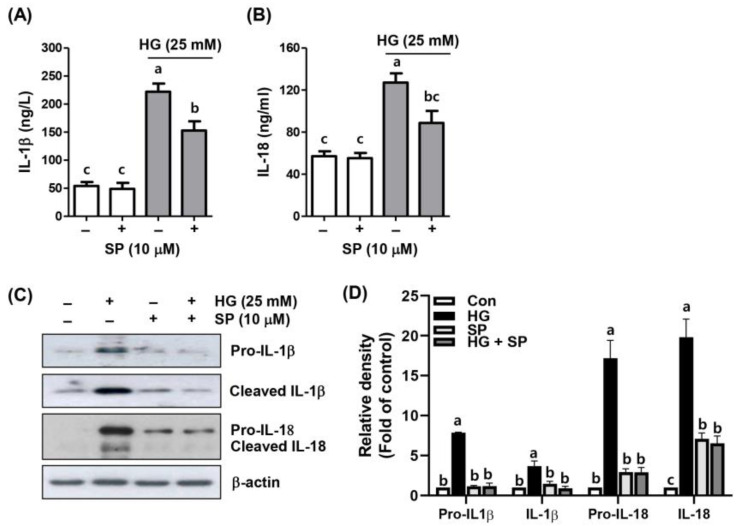
Attenuation of HG-induced cytokines and their protein expression by spermidine in ARPE-19 cells. (**A**,**B**) Concentration of interleukin (IL)-1β (**A**) and IL-18 (**B**) released in supernatant of cells exposed to HG with or without spermidine measured using enzyme-linked immunosorbent assay (ELISA) kits. (**C**,**D**) Levels of the proteins of interest were assessed by Western blotting (**C**), and the quantification results are shown (**D**). Numerical data are shown as the mean ± SD of three independent experiments. ^a–c^ Bars with different letters are significantly different at *p* < 0.05.

Next, we assessed the secretion of pro-inflammatory cytokines in the supernatants of ARPE-19 cells cultured under HG in the presence or absence of spermidine to explore the blocking effect of spermidine on the HG-promoted inflammatory response. Enzyme-linked immunosorbent assay (ELISA) results indicated that HG stimulation increased the inflammatory response, as revealed by enhanced levels of IL-1β and IL-18, but not in cells treated with spermidine alone (Figure 4A,B). Additionally, the protein expression was elevated under HG conditions (Figure 4C). However, after the spermidine intervention, the levels and protein expression of these inflammatory factors were increased by the HG and were markedly reduced compared to those in the HG group (Figure 4C,D). These results showed that spermidine counteracted HG-induced inflammation in ARPE-19 cells.

### 2.5. Spermidine Mitigated HG-Induced NF-κB Signaling Activation

**Figure 5:**
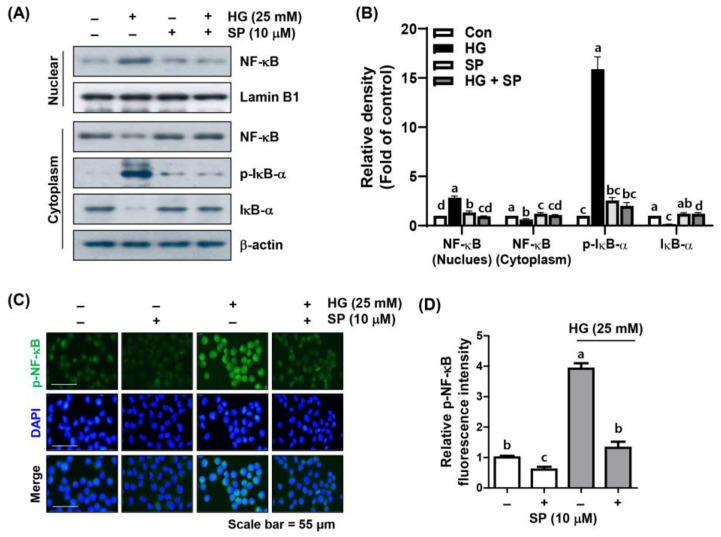
Inhibition of HG-induced nuclear factor kappa B (NF-κB) activation by spermidine in ARPE-19 cells. (**A**,**B**) Cells pre-treated with spermidine for 1 h were further cultured in a medium containing HG for another 1 h, and then Western blot analysis was conducted using nuclear and cytoplasmic fractions (**A**). The quantification results are shown (**B**). (**C**,**D**) Cellular localization of phosphorylated (p)-NF-κB p65 (green) in cells cultured under the same experimental setting observed by immunofluorescence (400×). Nuclei (blue) were counterstained with DAPI (**C**), and the quantification results are shown (**D**). Numerical data are shown as the mean ± SD of three independent experiments. ^a–d^ Bars with different letters are significantly different at *p* < 0.05.

Since NF-κB is a nuclear transcription factor that has an important role required for the transcription of inflammation-related factors, we verified whether the blocking of HG-promoted inflammation by spermidine is dependent on the NF-κB pathway. According to the results of immunoblotting and immunofluorescence presented in Figure 5, NF-κB protein levels in cells cultured under HG conditions significantly increased in the nuclear fraction rather than in the cytoplasmic fraction, and phosphorylated (p)-NF-κB was also preferentially expressed in the nucleus. Additionally, the expression levels of p-IκB-α increased in the cytoplasm, whereas the expression of IκB-α decreased. However, in the presence of spermidine, the nuclear localization of the HG-induced NF-κB, as well as the expression patterns of p-IκB-α and IκB-α, decreased, similar to that of the control group. These results suggest that spermidine alleviates HG-primed NF-κB activation, thereby reducing the associated inflammatory response.

### 2.6. Spermidine Attenuated HG-Induced NLRP3 Inflammasome Activation

**Figure 6:**
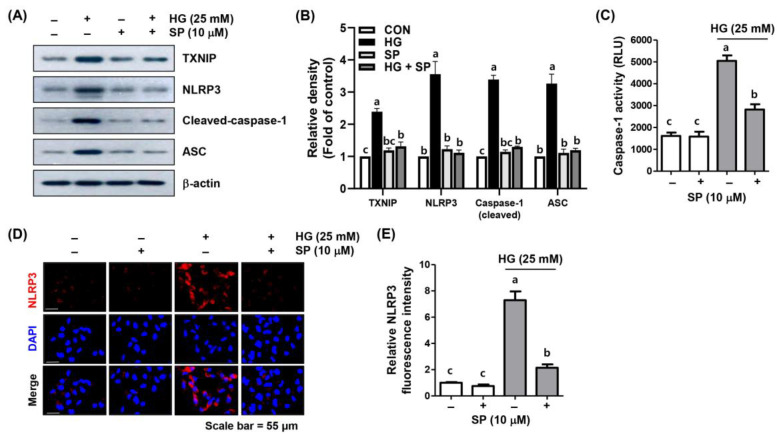
Suppression of HG-induced NLRP3 inflammasome by spermidine in ARPE-19 cells. (**A**,**B**) Cells pre-treated with spermidine for 1 h were further cultured in a medium containing HG for another 48 h, and then Western blot analysis was conducted (**A**). The quantification results are shown (**B**). (**C**) Caspase-1 activity was assessed with a fluorescence assay kit. (**D**,**E**) Cellular localization of NLRP3 (red) in cells cultured under the same experimental setting observed by immunofluorescence (400×). Nuclei (blue) were counterstained with DAPI (**D**). The quantification results are shown (**E**). Numerical data are presented as the mean ± SD of three independent experiments. ^a–c^ Bars with different letters are significantly different at *p* < 0.05.

NF-κB-induced NLRP3 inflammasome activation plays a critical role in the onset and development of DR, and the NLRP3 inflammasome is known to be a multiprotein complex that mediates pro-inflammatory cytokine secretion, which includes IL-1β and IL-18. Since spermidine attenuated the HG-induced secretion of IL-1β and IL-18, as well as NF-κB activation, we further investigated whether these inhibitory effects contributed to the blocking of the NLRP3 inflammasome pathway. Based on our immunoblotting results, the expression levels of TXNIP, an NLRP3 interacting molecule, and the NLRP3 inflammasome multiprotein complex molecules, ASC and caspase-1, were upregulated under HG conditions, whereas spermidine reduced these effects, similar to the control group (Figure 6A,B). To confirm these results, fluorescence and immunofluorescence assays were conducted to measure intracellular caspase-1 activity and NLRP3 expression levels. The results demonstrated that caspase-1 activity and NLRP3 levels were notably enhanced under HG conditions and were reduced by spermidine to levels similar to those in the control (Figure 6C–E).

## 3. Discussion

DR is a complication of diabetes that causes eye deterioration. Among various physiological markers, elevated glucose levels are pathogenic characteristics of DR. It is well documented that hyperglycemia promotes increased oxidative stress and inflammatory responses in DR [23]. Diabetes is accompanied by elevated free radical production and an impaired antioxidant defense system, indicating a critical contribution of ROS to the onset, progression, and pathological consequences of the disease. Mitochondrial ROS are considered a major endogenous cellular source of increased oxidative stress and are emerging as key risk factors for diabetic complications [24]. Several studies have demonstrated that antioxidants exert beneficial effects on the onset and progression of DR [24,25]. Spermidine alleviates oxidative stress by decreasing sensitivity to ROS [26]. In the current study, we assessed the protective effects of spermidine against oxidative stress and inflammation in RPE cells under HG conditions.

We treated RPE cells with HG (25 mM) to mimic hyperglycemia and measured both intracellular and mitochondrial ROS levels. Based on the results, both intracellular and mitochondrial ROS levels were markedly increased in RPE cells, confirming that hyperglycemic conditions are characterized by high oxidative stress. A previous study reported that HG increased the production of mitochondrial ROS, which is a main cause of hyperglycemic cellular dysfunction [27]. They reported that exogenous HG treatment to cells promoted an increase in cytosolic Ca^2+^, leading to mitochondrial fragmentation and ROS generation. Furthermore, HG induced an increase in antioxidant enzymes, such as catalase and glutathione peroxidase (GPx), in human umbilical vein endothelial cells, indicating that HG levels are associated with an increase in oxidative stress [28]. Although the antioxidant capacity and elevated ROS production were not investigated in the current study, antioxidant genes such as catalase, GPx, and superoxide dismutase could be suppressed, leading to ROS overwhelming the antioxidant capacity and resulting in a state of oxidative stress, which further contributes to the pathogenesis of DR. In addition, co-treatment with spermidine notably suppressed both the intracellular and mitochondrial ROS levels in our study. Although supraphysiological levels of spermidine may promote greater ROS production, optimal treatment with spermidine induces protective effects on intracellular oxidative stress levels.

**Figure 7:**
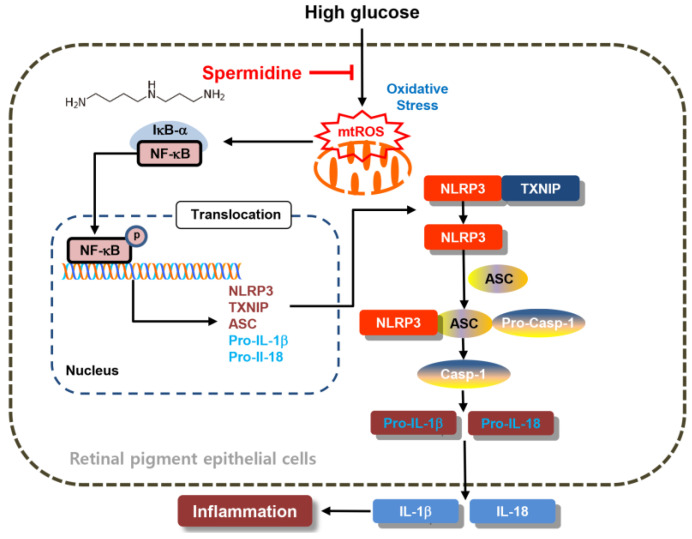
Schematic diagram of inhibitory effects of spermidine on mitochondrial ROS-mediated NF-κB/NLRP3 signaling activation, leading to alleviation of inflammation in hyperglycemic retinal pigment epithelial cells.

Elevated ROS production activates different signaling pathways. Typically, H_2_O_2_ activates the NF-κB signaling pathway, as demonstrated in different experimental models, including ARPE-19 cells [29,30]. In addition, upstream kinases that are oxidized by ROS can also activate the NF-κB signaling pathway. For example, this pathway can also be activated by serine/threonine Akt/protein kinase B [31]. H_2_O_2_ regulates IκB kinase (IKK)-dependent NF-κB activation through the NIK/IKK, phosphatidylinositol 3-kinase/phosphatase, and tensin homolog/Akt pathways [31]. Previous studies have shown that HG-induced oxidative stress promoted ROS production through the rapid activation of the mitogen-activated protein kinase, ERK1/2 [27], which is a well-known upstream regulator of NF-κB during stress responses. Similarly, HG conditions activated the ERK signaling pathway [32], which regulates NF-κB activation and its target genes’ expression. Furthermore, HG increased mRNA expression levels and the activation of toll-like receptor 4 [33,34], which also activates the NF-κB transcription factor. The NLRP3 inflammasome is a multiprotein complex that promotes inflammatory reactions within cells. NLRP3 becomes activated dependent on the NF-κB signaling pathway in hepatocytes [35]. As shown in our results, HG conditions upregulated the nuclear levels of NF-κB and its target NLRP3-associated TXNIP, as well as the NLRP3 multiprotein complex molecules, NLRP3, ASC, pro-IL-1β, and pro-IL18. However, spermidine reversed this pro-inflammatory condition and suppressed cytokine production and NLRP3 multiprotein complex molecules to levels comparable to those of the control group. Therefore, the current study showed that spermidine effectively suppresses NLRP3 signaling by suppressing upstream mitochondrial ROS-mediated NF-κB activation in hyperglycemic RPE cells (Figure 7).

## 4. Materials and Methods

### 4.1. Cell Culture and Treatment

ARPE-19 cells were acquired from the American Type Culture Collection (ATCC; Manassas, VA, USA) and cultured as described in the previous study [36]. In brief, cells were cultured in a Dulbecco’s Modified Eagle’s Medium/F-12 supplemented with fetal bovine serum (10%), penicillin (100 U/mL), and streptomycin (100 U/mL) at 37 °C under atmospheric conditions at 5% CO_2_. The cells were pre-treated with or without the indicated concentrations of spermidine and/or NAC for 1 h before inducing oxidative damage with glucose for 48 h.

### 4.2. Cell Proliferation Assay

Cell viability was observed under different treatment settings and investigated using an MTT assay (Sigma-Aldrich Co., St. Louis, MO, USA). The experimental procedures followed the manufacturer’s manual [37].

### 4.3. Apoptosis Assay

Apoptotic cells were assessed by flow cytometry with an Annexin V-fluorescein isothiocyanate (FITC)/propidium iodide (PI) apoptosis detection supply (BD Biosciences, San Jose, CA, USA). The experimental procedures followed the manufacturer’s manual [38].

### 4.4. Measurement of ROS Generation

ROS production in the total of the cells and mitochondria was detected using DCF-DA (Thermo Fisher Scientific, Waltham, MA, USA) and MitoSox (Molecular Probes, Eugene, OR, USA) fluorescence probes, respectively. The experimental procedures followed the previous study [39].

### 4.5. Analysis of Cytokine Levels

Following different treatments, cell supernatants were gathered, and IL-1β and IL-18 levels were examined using ELISA kits (R&D Systems Inc., Minneapolis, MN, USA). The experimental procedures followed the manufacturer’s manual and the previous study [40].

### 4.6. Western Blot Analysis

Cells were subject to lysis using a RIPA buffer or a nuclear and cytoplasmic protein extraction supply (Thermo Fisher Scientific), following the supplier’s instructions. After protein quantification, experimental procedures followed the previous study [41]. Antibodies were acquired from Thermo Fisher Scientific, Cell Signaling Technology Inc. (Beverly, MA, USA), Santa Cruz Biotechnology, Inc. (Dallas, TX, USA), and Abcam (Cambridge, UK). For all primary antibodies used in this study, see the Supplementary Information. Primary antibodies, sources, dilutions, and product information are listed in Table S1. Target proteins were detected using a chemiluminescence reagent (Thermo Fisher Scientific).

### 4.7. Immunofluorescence for NF-κB and NLRP3

Immunofluorescence analysis to detect phosphorylated (p)-NF-κB and NLRP3 in cells cultured for 1 or 48 h with or without spermidine was conducted following the previous study [42].

### 4.8. Caspase-1 Activity Measurement

Caspase-1 activity in the cell-free supernatants of HG-stimulated cells with or without spermidine was detected using a Caspase-1/ICE fluorescence assay kit (R&D Systems Inc.). The experimental procedures followed the manufacturer’s manual.

### 4.9. Statistical Analysis

Data were analyzed and visualized by one-way analysis of variance using Tukey’s post hoc test in GraphPad Prism version 8.4.2 (GraphPad Software Inc., La Jolla, CA, USA).

## 5. Conclusions

Our study demonstrated that spermidine was effective in suppressing NLRP3 inflammasome activation by downregulating NLRP3-associated and NLRP3-associated signaling complex molecules, such as TXNIP, NLRP3, ASC, and caspase-1, through mitochondrial ROS-mediated NF-κB activation in hyperglycemic RPE cells.

## Data Availability

The data presented in this study are available on request from the corresponding author.

## Supplementary Materials

The following supporting information can be downloaded at: https://www.mdpi.com/article/10.3390/ijms241310550/s1.

## References

[1] Tan T.E., Wong T.Y. (2023). Diabetic retinopathy: Looking forward to 2030. Front. Endocrinol..

[2] Trott M., Driscoll R., Pardhan S. (2022). Associations between diabetic retinopathy, mortality, disease, and mental health: An umbrella review of observational meta-analyses. BMC Endocr. Disord..

[3] Cheng Y., Ren T., Wang N. (2023). Biomechanical homeostasis in ocular diseases: A mini-review. Front. Public Health.

[4] Zhou Q., Yang L., Wang Q., Li Y., Wei C., Xie L. (2022). Mechanistic investigations of diabetic ocular surface diseases. Front. Endocrinol..

[5] Kang H., Yin N., Lyon H., Rupenthal I.D., Thakur S.S., Mugisho O.O. (2021). The influence of hyperglycemia on the safety of ultrasound in retinal pigment epithelial cells. Cell Biol. Int..

[6] Karthikkeyan G., Nareshkumar R.N., Aberami S., Sulochana K.N., Vedantham S., Coral K. (2018). Hyperglycemia induced early growth response-1 regulates vascular dysfunction in human retinal endothelial cells. Microvasc. Res..

[7] Ren J., Zhang S., Pan Y., Jin M., Li J., Luo Y., Sun X., Li G. (2022). Diabetic retinopathy: Involved cells, biomarkers, and treatments. Front. Pharmacol..

[8] Cunha-Vaz J. (2017). The blood-retinal barrier in the management of retinal disease: EURETINA award lecture. Ophthalmologica.

[9] Wang J., Li M., Geng Z., Khattak S., Ji X., Wu D., Dang Y. (2022). Role of oxidative stress in retinal disease and the early intervention strategies: A review. Oxid. Med. Cell. Longev..

[10] Dammak A., Huete-Toral F., Carpena-Torres C., Martin-Gil A., Pastrana C., Carracedo G. (2021). From oxidative stress to inflammation in the posterior ocular diseases: Diagnosis and treatment. Pharmaceutics.

[11] Kang Q., Yang C. (2020). Oxidative stress and diabetic retinopathy: Molecular mechanisms, pathogenetic role and therapeutic implications. Redox Biol..

[12] Li X., Fang P., Mai J., Choi E.T., Wang H., Yang X.F. (2013). Targeting mitochondrial reactive oxygen species as novel therapy for inflammatory diseases and cancers. J. Hematol. Oncol..

[13] Patergnani S., Bouhamida E., Leo S., Pinton P., Rimessi A. (2021). Mitochondrial oxidative stress and “Mito-Inflammation”: Actors in the diseases. Biomedicines.

[14] Naik E., Dixit V.M. (2011). Mitochondrial reactive oxygen species drive proinflammatory cytokine production. J. Exp. Med..

[15] Kuo C.Y., Maran J.J., Jamieson E.G., Rupenthal I.D., Murphy R., Mugisho O.O. (2022). Characterization of NLRP3 inflammasome activation in the onset of diabetic retinopathy. Int. J. Mol. Sci..

[16] Chen H., Zhang X., Liao N., Mi L., Peng Y., Liu B., Zhang S., Wen F. (2018). Enhanced expression of NLRP3 inflammasome-related inflammation in diabetic retinopathy. Investig. Ophthalmol. Vis. Sci..

[17] Loukovaara S., Piippo N., Kinnunen K., Hytti M., Kaarniranta K., Kauppinen A. (2017). NLRP3 inflammasome activation is associated with proliferative diabetic retinopathy. Acta Ophthalmol..

[18] Kinoshita T., Imamura R., Kushiyama H., Suda T. (2015). NLRP3 mediates NF-κB activation and cytokine induction in microbially induced and sterile inflammation. PLoS ONE.

[19] Wei Z.X., Cai L., Zhao X.M., Jiang X.R., Li X.L. (2022). Effects of spermidine on cell proliferation, migration, and inflammatory response in porcine enterocytes. Front. Biosci..

[20] Larqué E., Sabater-Molina M., Zamora S. (2007). Biological significance of dietary polyamines. Nutrition.

[21] Park I.H., Kim M.M. (2011). Inhibitory effect of spermidine with antioxidant activity on oxidative stress in human dermal fibroblasts. J. Life Sci..

[22] Chen Z., Lin C.X., Song B., Li C.C., Qiu J.X., Li S.X., Lin S.P., Luo W.Q., Fu Y., Fang G.B. (2020). Spermidine activates RIP1 deubiquitination to inhibit TNF-α-induced NF-κB/p65 signaling pathway in osteoarthritis. Cell Death Dis..

[23] Al-Kharashi A.S. (2018). Role of oxidative stress, inflammation, hypoxia and angiogenesis in the development of diabetic retinopathy. Saudi J. Ophthalmol..

[24] Nishikawa T., Araki E. (2007). Impact of mitochondrial ROS production in the pathogenesis of diabetes mellitus and its complications. Antioxid. Redox Signal..

[25] Yildirim Z., Uçgun N.I., Kiliç N., Gürsel E., Sepici-Dinçel A. (2007). Antioxidant enzymes and diabetic retinopathy. Ann. N. Y. Acad. Sci..

[26] Rider J.E., Hacker A., Mackintosh C.A., Pegg A.E., Woster P.M., Casero R.A. (2007). Spermine and spermidine mediate protection against oxidative damage caused by hydrogen peroxide. Amino Acids.

[27] Yu T., Jhun B.S., Yoon Y. (2011). High-glucose stimulation increases reactive oxygen species production through the calcium and mitogen-activated protein kinase-mediated activation of mitochondrial fission. Antioxid. Redox Signal..

[28] Ceriello A., dello Russo P., Amstad P., Cerutti P. (1996). High glucose induces antioxidant enzymes in human endothelial cells in culture. Evidence linking hyperglycemia and oxidative stress. Diabetes.

[29] Oliveira-Marques V., Marinho H.S., Cyrne L., Antunes F. (2009). Role of hydrogen peroxide in NF-kappaB activation: From inducer to modulator. Antioxid. Redox Signal..

[30] Lazzara F., Conti F., Platania C.B.M., Eandi C.M., Drago F., Bucolo C. (2021). Effects of vitamin D3 and meso-zeaxanthin on human retinal pigmented epithelial cells in three integrated in vitro paradigms of age-related macular degeneration. Front. Pharmacol..

[31] Kane Kane L.P., Shapiro V.S., Stokoe D., Weiss A. (1999). Induction of NF-kappaB by the Akt/PKB kinase. Curr. Biol..

[32] Lazzara F., Fidilio A., Platania C.B.M., Giurdanella G., Salomone S., Leggio G.M., Tarallo V., Cicatiello V., De Falco S., Eandi C.M. (2019). Aflibercept regulates retinal inflammation elicited by high glucose via the PlGF/ERK pathway. Biochem. Pharmacol..

[33] Lazzara F., Longo A.M., Giurdanella G., Lupo G., Platania C.B.M., Rossi S., Drago F., Anfuso C.D., Bucolo C. (2022). Vitamin D3 preserves blood retinal barrier integrity in an in vitro model of diabetic retinopathy. Front. Pharmacol..

[34] Dasu M.R., Devaraj S., Zhao L., Hwang D.H., Jialal I. (2008). High glucose induces toll-like receptor expression in human monocytes: Mechanism of activation. Diabetes.

[35] Boaru S.G., Borkham-Kamphorst E., Van de Leur E., Lehnen E., Liedtke C., Weiskirchen R. (2015). NLRP3 inflammasome expression is driven by NF-κB in cultured hepatocytes. Biochem. Biophys. Res. Commun..

[36] Hong S.H., Park C., Hwangbo B., Bang E.J., Kim S.O., Shim J.H., Park S.H., Lee H., Leem S.H., Kim G.Y. (2022). Activation of heme oxygenase-1 is involved in the preventive effect of honokiol against oxidative damage in human retinal pigment epithelial cells. Biotechnol. Bioprocess. Eng..

[37] Lee S.H., Tsutsui M., Matsunaga A., Oe T. (2023). Lipid hydroperoxide-derived insulin resistance and its inhibition by pyridoxamine in skeletal muscle cells. Toxicol. Res..

[38] Choi Y.H. (2022). Tacrolimus induces apoptosis in leukemia Jurkat cells through inactivation of the reactive oxygen species-dependent phosphoinositide-3-kinase/Akt signaling pathway. Biotechnol. Bioprocess Eng..

[39] Kim M.Y., Bang E., Hwangbo H., Ji S.Y., Kim D.H., Lee H., Park C., Hong S.H., Kim G.Y., Choi Y.H. (2023). Diallyl trisulfide inhibits monosodium urate-induced NLRP3 inflammasome activation via NOX3/4-dependent mitochondrial oxidative stress in RAW 264.7 and bone marrow-derived macrophages. Phytomedicine.

[40] Hwangbo H., Ji S.Y., Kim M.Y., Kim S.Y., Lee H., Kim G.Y., Kim S., Cheong J., Choi Y.H. (2021). Anti-inflammatory effect of auranofin on palmitic acid and LPS-induced inflammatory response by modulating TLR4 and NOX4-mediated NF-κB signaling pathway in RAW 264.7 macrophages. Int. J. Mol. Sci..

[41] Lee J.K., Choi W.S., Song J.Y., Kwon O.S., Lee Y.J., Lee J.S., Lee S., Choi S.R., Lee C.H., Lee J.Y. (2023). Anti-inflammatory effects of *Athyrium yokoscense* extract via inhibition of the Erk1/2 and NF-κB pathways in bisphenol A-stimulated A549 cells. Toxicol. Res..

[42] Lee H., Park C., Kwon D.H., Hwangbo H., Kim S.Y., Kim M.Y., Ji S.Y., Kim D.H., Jeong J.W., Kim G.Y. (2021). Schisandrae Fructus ethanol extract attenuates particulate matter 2.5-induced inflammatory and oxidative responses by blocking the activation of the ROS-dependent NF-κB signaling pathway. Nutr. Res. Pract..

