# Supplementary material for: Spermidine Attenuates High Glucose-Induced Oxidative Damage in Retinal Pigment Epithelial Cells by Inhibiting Production of ROS and NF-κB/NLRP3 Inflammasome Pathway
Source: Int J Mol Sci. 2023 Jun 23;24(13):10550. doi: 10.3390/ijms241310550 (PMC10341649; doi:10.3390/ijms241310550)
Supplement: Supplementary file 1 [file ijms-24-10550-s001.zip › ijms-2455788-supplementary.pdf]

Supplementary information

Table S1. List of antibodies

Primary antibody	Dilution	Source	Manufacturer	Catalog No.
**Pro-IL-1β**	1:1000	rabbit	Santa Cruz	sc-7884
**IL-1β**	1:1000	rabbit	Santa Cruz	sc-7884
**IL-18**	1:1000	rabbit	Abcam	ab71495
**NF-κB**	1:1000	mouse	Cell signaling	6956P
**p-IκB-α**	1:1000	mouse	Thermo Fisher Scientific	MA5-15224
**IκB-α**	1:1000	mouse	Abcam	ab97783
**TXNIP**	1:1000	rabbit	Abcam	ab188865
**NLRP3**	1:1000	rabbit	Abcam	Ab263899
**Cleaved-caspase-1**	1:1000	rabbit	Santa Cruz	sc-622
**ASC**	1:1000	rabbit	Thermo Fisher Scientific	10500-1-AP
**Lamin B1**	1:1000	goat	Santa Cruz	sc-6216
**β-actin**	1:1000	mouse	BioWorld	BS6007M
